# DNA hypomethylation of the Krüppel-like factor 11 (*KLF11*) gene promoter: a putative biomarker of depression comorbidity in panic disorder and of non-anxious depression?

**DOI:** 10.1007/s00702-020-02216-9

**Published:** 2020-06-10

**Authors:** Leonie Kollert, Miriam A. Schiele, Christiane Thiel, Andreas Menke, Jürgen Deckert, Katharina Domschke

**Affiliations:** 1 Department of Psychiatry, Psychosomatics and Psychotherapy, University of Würzburg, Würzburg, Germany; 2 Department of Psychiatry and Psychotherapy, Medical Center-University of Freiburg, Faculty of Medicine, University of Freiburg, Freiburg, Germany; 3 Center for Basics in Neuromodulation, Faculty of Medicine, University of Freiburg, Freiburg, Germany

**Keywords:** KLF11, TIEG2, Epigenetics, Panic disorder, Major depressive disorder, TGFB-inducible early growth response protein 2

## Abstract

Panic disorder (PD) is one of the most common anxiety disorders and often occurs comorbidly with major depressive disorder (MDD). Altered methylation of the monoamine oxidase A (*MAOA*) gene has been implicated in the etiology of both PD and MDD. The Krüppel-like factor 11 (KLF11; alias TIEG2), an activating transcription factor of the *MAOA* gene, has been found to be increased in MDD, but has not yet been investigated in PD. In an effort to further delineate the effects of the KLF11–MAOA pathway in anxiety and affective disorders, *KLF11* promoter methylation was analyzed via pyrosequencing of sodium bisulfite-treated DNA isolated from human peripheral blood in two independent samples of PD patients with or without comorbid MDD in a case–control design (sample 1: *N* = 120) as well as MDD patients with and without anxious depression (sample 2: *N* = 170). Additionally, in sample 1, *KLF11* methylation was correlated with Beck Depression Inventory (BDI-II) scores. No overall association of *KLF11* promoter methylation with PD was detected. However, PD patients with comorbid MDD showed significant hypomethylation relative to both healthy controls (*p* = 0.010) and PD patients without comorbid MDD (*p* = 0.008). Furthermore, *KLF11* methylation was negatively correlated with BDI-II scores in PD patients (*p* = 0.013). MDD patients without anxious features showed nominally decreased *KLF11* methylation in comparison to MDD patients with anxious depression (*p* = 0.052). The present results suggest *KLF11* promoter hypomethylation as a potential epigenetic marker of MDD comorbidity in PD or of non-anxious depression, respectively, possibly constituting a differential pathomechanism in anxiety and mood disorders.

## Introduction

Panic disorder (PD) is one of the most common anxiety disorders, with lifetime prevalence rates of 2.1–4.7% (Baxter et al. 2014) and a 12-month prevalence of 1.8% (Goodwin et al. 2005). PD is characterized by sudden, unexpected and recurrent panic attacks, i.e., episodes of extreme fear (American Psychiatric Association 2013). PD is often comorbid with major depressive disorder (MDD; Gorman and Coplan 1996): Lifetime prevalence rates of comorbid MDD in PD are estimated at about 30–40% (Fava et al. 2000; Lamers et al. 2011; Kessler et al. 2015), similar to the lifetime prevalence rates of comorbid MDD in the group of anxiety disorders as a whole (Kessler et al. 2006).

The monoamine oxidase A (MAOA)—metabolizing catecholamines such as norepinephrine and serotonin and being a target of potent antidepressants such as tranylcypromine—has been suggested as a key player in the pathogenesis of anxiety and mood disorders (for review see Ziegler and Domschke 2018). On an epigenetic level, *MAOA* hypomethylation has been reported in patients with PD (Domschke et al. 2012; Ziegler et al. 2016), acrophobia (Schiele et al. 2018), and MDD (Melas et al. 2013; Melas and Forsell 2015). Moreover, *MAOA* hypomethylation has been observed to be predictive of response to pharmacological treatment in MDD (Domschke et al. 2012) and to be modifiable by psychotherapeutic interventions in anxiety disorders (Ziegler et al. 2016; Schiele and Domschke 2018; Schiele et al. 2018; for review see Schiele et al. 2020).

Recent studies have highlighted the Krüppel-like factor 11 (KLF11; alias TGFB-Inducible Early Growth Response Protein 2 [TIEG2]) as a novel transcriptional activator of *MAOA* gene expression (for review see Duncan et al. 2012). KLF11 is a member of the Sp/KLF family of zinc finger transcription factors and regulates the transcription of neuronal genes by binding to distinct sequences within the target gene promoter region, triggering RNA polymerase II (Pol II)-mediated transcriptional initiation (Ou et al. 2004; Harris et al. 2015). The *MAOA* promoter contains four Sp1-binding sites which have been reported to be a target of KLF11. Accordingly, KLF11 transfection has been observed to result in a twofold increase in *MAOA* mRNA expression (Grunewald et al. 2012). Further support for a KLF11–MAOA pathway emerges from a study showing elevated KLF11 protein levels correlating with increased MAOA levels in postmortem brain samples of MDD patients (Harris et al. 2015).

Given these close links between KLF11 and MAOA function, the *KLF11* (*TIEG2*) gene on chromosome 2p25.1 might constitute a prime, yet uninvestigated candidate gene in the panic disorder/MDD spectrum. Thus, in the present study, we for the first time examined *KLF11* DNA methylation levels in (1) patients with panic disorder in a case–control design taking into account comorbidity with MDD, and (2) in an independent sample of patients with MDD by comparing symptom subtypes (i.e., anxious vs. non-anxious depression) in an effort to for the first time explore the role of KLF11 on an epigenetic level in anxiety and affective disorders.

## Materials and methods

### Samples

*Sample 1* consisted of 60 Caucasian (for at least two preceding generations) patients with PD (47 females; age [mean ± SD]: 34.28 ± 9.49 years) with (*N* = 28, 46.6%) or without agoraphobia, and 60 healthy controls matched by age (mean ± SD: 34.30 ± 9.25 years; *t* = 0.01, *df* = 118, *p* = 0.992) and sex (47 females; Χ^2^ = 0.0, *df* = 1, *p* = 1.0). PD diagnosis was confirmed by experienced clinical psychologists on the basis of a clinical interview according to DSM-IV criteria (SCID-I; Wittchen et al. 1997). The presence of current comorbid axis I diagnoses other than bipolar disorder, psychotic disorders, current alcohol dependence, current abuse of or dependence on benzodiazepines and other psychoactive substances was tolerated if PD was the primary diagnosis (MDD: *N* = 28; social anxiety disorder: *N* = 3; specific phobias: *N* = 2). Further exclusion criteria were current or past internal or neurological somatic illnesses, intake of any somatic medication, illegal drugs including cannabis (assessed by urine toxicology), pregnancy and excessive alcohol (> 15 glasses of alcohol per week) or nicotine (> 20 cigarettes per day) consumption. Thirty-four (56.7%) patients were on stable psychiatric medication with selective serotonin reuptake inhibitors (SSRIs, *N* = 18), serotonin-norepinephrine reuptake inhibitors (SNRIs, *N* = 5), a noradrenergic and specific serotonergic antidepressant (NaSSA, *N* = 7), tricyclic antidepressants (TCAs, *N* = 7), pregabaline (*N* = 2), quetiapine (*N* = 2), or zopiclone (*N* = 1). No patient received monoamine oxidase inhibitors or valproate. Smoking status was assessed in the patient (smokers: *N* = 19) and control groups (*N* = 18). In the control group, absence of current and/or lifetime DSM-IV mental axis I disorders was assessed using the Mini International Neuropsychiatric Interview (M.I.N.I.; Sheehan et al. 1998). The severity of depressive symptoms in patients was assessed using the 21-item Beck Depression Inventory (BDI-II; Hautziger et al. 2009). All participants were recruited at the Department of Psychiatry, University of Würzburg, Germany, within the Collaborative Research Centre SFB-TRR-58 ‘Fear, Anxiety, Anxiety Disorders’, project C02.

*Sample 2* comprised 170 Caucasian patients diagnosed with major depressive disorder (MDD; 98 females; age [mean ± SD]: 44.61 ± 14.86 years) with (≥ 7 on the anxiety/somatization factor of the Hamilton Depression Rating Scale; cf. Fava et al. 2008; Domschke et al. 2010; *N* = 99, 58.24%) or without anxious depression. MDD diagnosis was determined by experienced clinical psychologists or psychiatrists based on medical records and a structured clinical interview according to DSM-IV criteria (SCID-I; Wittchen et al. 1997). Exclusion criteria were current obsessive–compulsive disorder, schizoaffective disorder, psychosis or dementia, and/or presence of substance abuse disorder or eating disorder currently or within the last 10 years. Smoking status was assessed in the overall sample (smokers: *N* = 52, 30.59%). *N* = 135 (79.4%) patients received psychiatric medication with selective serotonin reuptake inhibitors (SSRIs, *N* = 48), serotonin-norepinephrine reuptake inhibitors (SNRIs, *N* = 48), a noradrenergic and specific serotonergic antidepressant (NaSSA, *N* = 36), tricyclic antidepressants (TCAs, *N* = 26), a noradrenaline and dopamine reuptake inhibitor (NDRI, *N* = 6), neuroleptics (*N* = 33), anticonvulsants (*N* = 12), benzodiazepines (*N* = 13), lithium (*N* = 7), or tianeptine (*N* = 1). No patient received monoamine oxidase inhibitors or valproate. All participants were recruited at the Department of Psychiatry, University of Würzburg, Germany.

This study was approved by the ethics committee of the University of Würzburg, Germany, and was conducted according to the ethical principles of the Helsinki Declaration. Written informed consent was obtained from all participants.

### *KLF11* DNA methylation analysis

Venous blood samples were collected from all participants using standardized EDTA tubes. DNA extractions from frozen whole blood were accomplished using either FlexiGene^®^ DNA Kit (Qiagen, Hilden, Germany) or a standardized salting out procedure (Miller et al. 1988). After pre-analysis of a 313 bp-long *KLF11* promoter region (chr2:10,182,891–10,183,204; GRCh37/hg19 Assembly, UCSC Genome Browser) by Sanger sequencing (LGC Genomics, Berlin, Germany) according to previous studies (e.g., Domschke et al. 2012; Ziegler et al. 2016; Schiele et al. 2018, 2019, 2020), a 72 bp amplicon containing part of the *KLF11* promoter (five CpGs) upstream of exon I (chr2:10,183,094–10,183,166; GRCh37/hg19 Assembly, UCSC Genome Browser) was chosen for further DNA methylation analyses, given that only these five CpG sites showed a considerable variance in their mean methylation level and thus seemed to be suitable for subsequent DNA methylation analyses by pyrosequencing.

Aliquots (500 ng) of isolated genomic DNA were treated with sodium bisulfite using the EpiTect^®^ 96 Bisulfite Kit (Qiagen, Hilden, Germany) according to the manufacturer’s instructions and processed in randomized order to avoid possible batch effects. Non-methylated and fully methylated EpiTect^®^ PCR Control DNA Sets (Qiagen, Hilden, Germany) were used as a control for successful bisulfite conversion.

The 72 bp amplicon (10,183,094–10,183,166; GRCh37/hg19 Assembly, UCSC Genome Browser) was amplified via PCR according to a standard pyrosequencing PCR protocol (Qiagen, Hilden, Germany). The obtained DNA strand was sequenced and quantitatively analyzed by pyrosequencing using PyroMark^®^ Q96 ID System (Qiagen, Hilden, Germany). All samples were tested in duplicate to check for run variability resulting in a mean methylation score for each CpG site as well as an individual standard deviation (SD) for each duplicate. For quality control, SD > 0.1 of each duplicate was used as exclusion criterion. Additionally, outliers (≥ 3*SD from mean methylation of respective CpG site) were defined as second exclusion criterion. In each sample, *N* = 6 participants had to be excluded from the reported analyses according to these criteria.

The obtained electropherograms were robustly readable for all five CpG sites, and CpG sites were numbered according to their GRCh37/hg19 Primary Assembly position: CpG1 = chr2:10,183,094; CpG2 = chr2:10,183,106; CpG3 = chr2:10,183,115; CpG4 = chr2:10,183,125; CpG5 = chr2:10,183,140 (all genomic locations according to GRCh37/hg19 Assembly, UCSC Genome Browser).

### Statistical analysis

Differences in dimensional sample characteristics were tested by means of independent samples *t* tests, and differences in categorical variables by means of Chi-square tests. *KLF11* methylation (average methylation, single CpG sites) between PD patients and controls (sample 1) and between MDD patients with anxious and non-anxious depression (sample 2), respectively, was compared by means of non-parametric Kruskal–Wallis test. Correlations between *KLF11* methylation and BDI-II scores in sample 1 were evaluated by means of Spearman correlations (*ρ*). Since no associations between average *KLF11* methylation and sex (sample 1: *t*_112_ = 1.056, *p* = 0.293; sample 2: *t*_162_ = − 0.137, *p* = 0.891), age (sample 1: *r* = 0.156; *p* = 0.097; sample 2: *r* = 0.054; *p* = 0.495), smoking status (sample 1: *t*_112_ = 0.745, *p* = 0.458; sample 2: *t*_161_ = 1.213; *p* = 0.227), or medication intake (sample 1: *t*_57_ = 1.384, *p* = 0.172; sample 2: *t*_162_ = − 0.123, *p* = 0.902) were found in either sample, analyses were not controlled for these variables. The significance level was set at *p* ≤ 0.05. Given the present proof-of-concept approach and high correlation between single CpG sites (data not shown), no correction for multiple testing was applied when analyzing single CpG sites and average *KLF11* methylation (cf. Schiele et al. 2018).

## Results

### Sample 1

**Fig. 1:**
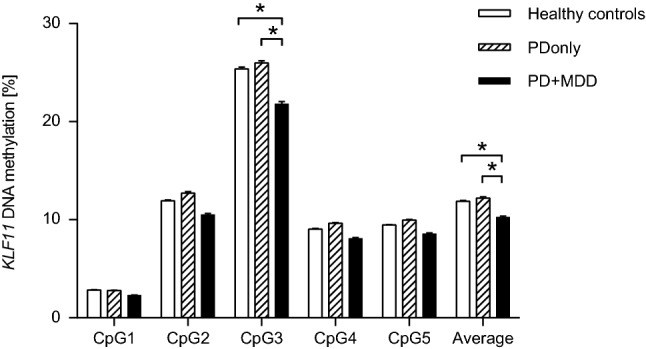
*KLF11* DNA methylation in panic disorder patients with or without comorbid major depressive disorder and matched healthy controls. *PD* panic disorder, *MDD* major depressive disorder, *Controls* healthy controls without any anxiety disorder, *PDonly* PD patients without comorbid MDD, *PD + MDD* PD patients with comorbid MDD; *p ≤ 0.05

In the overall case–control comparison between PD patients and healthy controls, no significant differences in average methylation or in DNA methylation at single CpG sites was discerned (see Table 1). Secondary analyses comparing PD patients with and without comorbid MDD indicated significant group differences for average methylation (*p* = 0.049) and methylation at CpG3 (*p* = 0.041). Follow-up tests revealed significantly decreased methylation in PD patients with comorbid MDD (PD + MDD; *N* = 27) regarding average *KLF11* methylation (*p* = 0.008) and CpG site 3 methylation (*p* = 0.006) compared to PD patients without comorbid MDD as well as compared to healthy controls, again regarding average methylation (*p* = 0.010) and methylation at CpG site 3 (*p* = 0.006) (see Table 2, Fig. 1). Conversely, PD patients without comorbid MDD diagnosis (PDonly; *N* = 30) did not differ from healthy controls with regard to *KLF11* average methylation and methylation at single CpG sites (all *p* > 0.05; see Table 2).

**Table 1:** *KLF11* methylation levels in PD patients and matched healthy controls

CpG	Sample 1^a^		
	PD patients (mean ± SE) *N* = 57	Controls (mean ± SE) *N* = 57	Statistics^b^
Average	11.29 ± 0.45	11.88 ± 0.64	*z* = − 0.25; *p* = 0.803
1	2.55 ± 0.14	2.83 ± 0.21	*z* = − 0.60; *p* = 0.552
2	11.67 ± 0.55	11.93 ± 0.65	*z* = − 0.10; *p* = 0.919
3	24.01 ± 0.91	25.38 ± 1.30	*z* = − 0.33; *p* = 0.740
4	8.90 ± 0.38	9.04 ± 0.46	*z* = − 0.00; *p* = 0.998
5	9.29 ± 0.35	9.46 ± 0.39	*z* = − 0.06; *p* = 0.956

^a^*PD* panic disorder

^b^*p *values from non-parametric Mann–Whitney *U* test are reported with average DNA methylation or methylation at the respective single CpG sites as dependent variable and group (PD patients vs. healthy controls). Mean methylation given in %; *SE* standard error of the mean

**Table 2:** *KLF11* methylation levels in PD patients with and without comorbid MDD and matched healthy controls

CpG	Sample 1^a^						
	Pdonly (mean ± SE) *N* = 30	PD + MDD (mean ± SE) *N* = 27	Controls (mean ± SE) *N* = 57	Statistics^b^	PDonly vs. controls^c^	PD + MDD vs. controls	PDonly vs. PD + MDD
Average	12.22 ± 0.61	10.25 ± 0.62	11.88 ± 0.63	H = 6.04; *p* = 0.049*	*z* = − 1.94; *p* = 0.053	*z* = − 2.73 *p* = 0.010**	*z* = − 2.65; *p* = 0.008**
1	2.79 ± 0.20	2.29 ± 0.18	2.84 ± 0.21	H = 4.35; *p* = 0.113	–	–	–
2	12.72 ± 0.76	10.51 ± 0.73	11.93 ± 0.65	H = 5.06; *p* = 0.080	–	–	–
3	25.99 ± 1.22	21.81 ± 1.25	25.38 ± 1.30	H = 6.39; *p* = 0.041*	*z* = − 1.93; *p* = 0.054	*z* = − 2.56; *p* = 0.006**	*z* = − 2.73; *p* = 0.006**
4	9.63 ± 0.51	8.09 ± 0.54	9.04 ± 0.46	H = 5.26; *p* = 0.072	–	–	–
5	9.95 ± 0.47	8.56 ± 0.49	9.46 ± 0.39	H = 5.59; *p* = 0.061	–	–	–

^a^*PD* panic disorder, *MDD* major depressive disorder

^b^*p* values from non-parametric Kruskal–Wallis test are reported with average DNA methylation or methylation at the respective single CpG sites as dependent variable and group (healthy controls vs. PDonly patients vs. PD + MDD patients)

^c^*z* and *p *values from post hoc tests (Mann–Whitney *U* test) are reported. Bold: significant results: *significant at *p* ≤ 0.05; **significant at *p* ≤ 0.01; mean methylation given in %; *SE* standard error of the mean

**Fig. 2:**
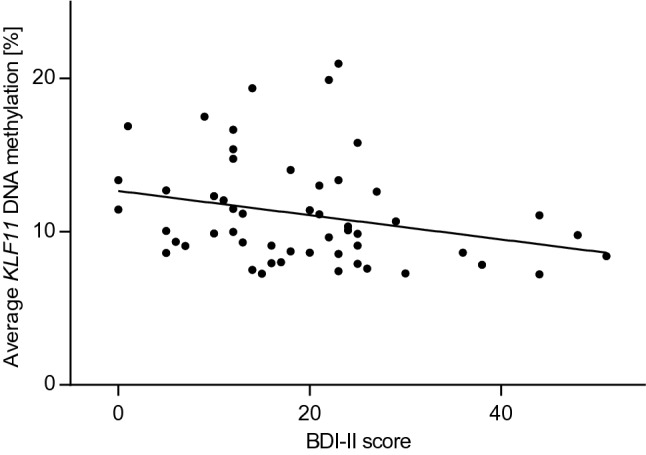
*KLF11* DNA methylation and dimensional depression in panic disorder. Negative correlation between average *KLF11* DNA methylation and Beck Depression Inventory (BDI-II) scores in 54 patients with panic disorder (*ρ* = −0.336, *p* = 0.013)

On a dimensional level, a negative correlation of *KLF11* average methylation with BDI-II scores was observed in the patient group (*ρ* = − 0.336; *p* = 0.013; see Fig. 2).

### Sample 2

**Fig. 3:**
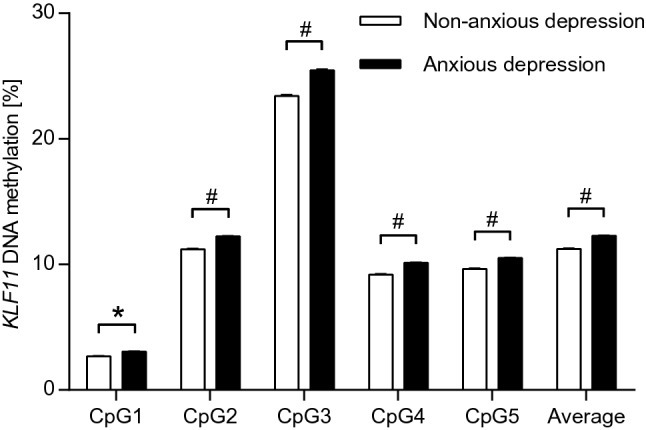
*KLF11* DNA methylation in MDD patients with or without anxious depression. MDD: major depressive disorder; anxious depression: 98 patients with anxious depression (anxiety/somatization factor of the Hamilton Depression Rating Scale ≥ 7); non-anxious depression: 66 patients without anxious depression (anxiety/somatization factor of the Hamilton Depression Rating Scale < 7); ^#^*p* ≤ 0.1; *p ≤ 0.05

Significant differences in *KLF11* DNA methylation were observed between MDD patients with and without anxious depression at CpG site 1 (*p* = 0.041) and, on a nominally significant level, with regard to average methylation (*p* = 0.052) and methylation at CpG sites 2–4 (*p* = 0.053-0.081; see Table 3; Fig. 3), with patients with non-anxious depression showing decreased *KLF11* methylation.

**Table 3:** *KLF11* DNA methylation in MDD patients with or without anxious depression

CpG	Sample 2^a^		
	Anxious depression (mean ± SE) *N* = 98	Non-anxious depression (mean ± SE) *N* = 66	Statistics^b^
Average	12.28 ± 0.37	11.23 ± 0.41	*z* = − 1.95; *p* = 0.052
1	3.07 ± 0.12	2.70 ± 0.13	*z* = − 2.04; *p* = 0.041*
2	12.23 ± 0.41	11.20 ± 0.46	*z* = − 1.75; *p* = 0.081
3	25.46 ± 0.70	23.41 ± 0.80	*z* = − 1.75; *p* = 0.079
4	10.13 ± 0.35	9.18 ± 0.38	*z* = − 1.77; *p* = 0.077
5	10.51 ± 0.31	9.64 ± 0.33	*z* = − 1.94; *p* = 0.053

^a^*MDD* major depressive disorder, *anxious depression* 98 patients with anxious depression (anxiety/somatization factor of the Hamilton Depression Rating Scale ≥ 7), *non-anxious depression* 66 patients without anxious depression (anxiety/somatization factor of the Hamilton Depression Rating Scale < 7).

^b^*p* values from non-parametric Mann–Whitney *U* test are reported with average DNA methylation or methylation at the respective single CpG sites as dependent variable and group (anxious vs. non-anxious MDD patients)

Bold: significant results: *significant at *p* ≤ 0.05; mean methylation given in %; *SE* standard error of the mean

## Discussion

In the present study, patients with PD and comorbid MDD displayed hypomethylation of the *KLF11* (*TIEG2*) promoter region in comparison to a matched healthy control group as well as to PD patients without comorbid MDD. Correspondingly, a negative correlation of *KLF11* DNA methylation with dimensional symptoms of depression (as evaluated via the BDI-II) was observed in the PD patient group. Furthermore, in an independent sample of MDD patients, MDD patients without anxious features showed nominally significantly decreased *KLF11* methylation levels as compared to MDD patients with anxious depression.

The present results for the first time demonstrating *KLF11* promoter hypomethylation—presumably leading to increased *KLF11* gene expression (cf. Suzuki and Bird 2008) and, consequently, an overexpression of the *MAOA* gene (Grunewald et al. 2012)—in depression-related phenotypes are in line with a previous finding of increased KLF11 and MAOA levels in MDD patients compared to healthy controls (Harris et al. 2015) and with studies reporting *MAOA* hypomethylation in female patients with MDD (Melas et al. 2013; Melas and Forsell 2015). The present findings, however, do not support a role of *KLF11* methylation in PD. Thus, against the theoretical background of a common biological trunk shared by anxiety and mood disorders, *KLF11* methylation could be instrumental in guiding the differentiation of the clinical phenotype toward depression- rather than panic-related symptoms.

Interestingly, the results of this study mirror a recently published finding of serotonin transporter gene (*SLC6A4*) promoter hypermethylation in PD patients with comorbid MDD relative to healthy controls, but not in PD per se (Schiele et al. 2019). Consequently, future studies might want to explore the biochemical relationship between KLF11 and SLC6A4 function in the context of MDD, especially given that the *SLC6A4* promotor region has been shown to contain Sp1 binding sites (Bengel et al. 1997; Heils et al. 1998), which might be targeted by KLF11 (cf. Grunewald et al. 2012).

The present findings should be interpreted in light of some limitations and might inspire future research efforts. For example, replication in independent, larger samples is warranted due to the relatively small group sizes of PD patients with and without comorbid depression, or MDD patients with and without anxious features, respectively, to confirm *KLF11* hypomethylation as a potential selective differential diagnostic marker of MDD comorbidity in PD or non-anxious MDD. Given that MDD is highly comorbid not only with PD but also with other anxiety and mental disorders, the potential of *KLF11* methylation status as a diagnostic marker to separate MDD diagnosis from other diagnoses should be explored in future studies. This would require a direct comparison with other comorbidity profiles, as well as comorbidity of MDD within other anxiety or mental disorders and a direct comparison of a PD sample with an MDD sample (i.e., PD with MDD vs. MDD only, PD without MDD vs. MDD only). Additionally, since no control group was available for the MDD sample (sample 2), the observed differences of *KLF11* methylation in MDD patients with or without anxious depression cannot be clearly ascribed to the effects of anxious symptoms. Moreover, longitudinal studies would help to elucidate whether the observed *KLF11* hypomethylation constitutes a trait or state marker of MDD. Despite the fact that in the present study PD patients and controls were matched for sex, age and smoking status, additional potentially confounding factors such as environmental exposure (cf. Domschke et al. 2012) cannot be excluded. Therefore, investigation of the impact of life events on *KLF11* methylation levels applying an (epi)gene-environment approach would pose a promising future research direction, particularly given previous evidence for a strong influence of stress on KLF11 expression (Grunewald et al. 2012; Harris et al. 2015). In addition, although no statistically significant influence of medication status on *KLF11* methylation was observed in either sample, potentially confounding effects of psychiatric medication on *KLF11* methylation cannot be fully excluded given that 57% (sample 1) or 79% (sample 2), respectively, of patients were on stable medication at the time of testing. Also, next to the differential effects of *KLF11* methylation on categorical disorder phenotypes, future studies may want to probe the association of *KLF11* methylation with symptom subtypes/subgroups as well as with disorder-specific intermediate phenotypes, and differential DNA methylation markers of panic disorder and MDD should be explored on an epigenome-wide level by means of epigenome-wide association studies (EWAS) (cf. Shimada-Sugimoto et al. 2017; Iurato et al. 2017; Shimada et al. 2018; Ziegler et al. 2019). Although pyrosequencing is currently considered the gold standard of DNA methylation analysis with a sensitivity to reliably detect differences as small as 5% (e.g., Dejeux et al. 2009; Migheli et al. 2013; Poulin et al. 2018), low methylation percentages (< 10%) may be confounded by unsystematic noise. In the present study, CpGs 2 and 3 achieved average methylation levels > 10% or > 20%, respectively, and might therefore constitute regions of particular interest for further analyses. For instance, analysis of a restricted target region of CpGs 2 and 3 utilizing the transcription factor-binding site prediction tool PROMO (Messeguer et al. 2002) identified the binding site of transcription factor TFI-II, a ubiquitously expressed protein, involved in growth factor signaling (Roy et al. 2007), to be located at CpG3, thus constituting a putative transcription factor whose binding affinity at this region could be affected by DNA methylation. Thus, future functional approaches may want to experimentally confirm a differential influence of *KLF11* methylation on TFI-II binding. Finally, DNA methylation was measured in peripheral blood samples. Given that epigenetic processes can act in a cell type-specific way, a cofounding effect due to potential variation in white blood cell composition between patients and controls cannot be excluded. Furthermore, epigenetic analyses in peripheral biomaterial such as blood does not allow for direct conclusions regarding methylation levels in brain tissue. Nevertheless, animal studies intraindividually comparing peripheral and central methylation or studies comparing peripheral methylation with central activity of the respective metabolite in humans using positron emission tomography (PET) have provided some evidence for DNA methylation levels measured in blood as a proxy for central processes (e.g., Murphy et al. 2005; Nohesara et al. 2011; Shumay et al. 2012). Also, when performing an *in silico* analysis of the correlation between blood and brain DNA methylation levels at the investigated *KLF11* region using the online available “Blood Brain DNA Methylation Comparison Tool” (Hannon et al. 2015), peripheral DNA methylation of both CpG1 (cg22541755) and CpG3 (cg20702913) was positively correlated with prefrontal cortex methylation (*r* ≥ 0.332, *p* ≤ 0.004). Analysis of further *KLF11* CpGs was precluded by the fact that only CpGs 1 and 3 are available in this search tool based on Illumina 450 K array data.

In conclusion, the present pilot data suggest *KLF11* promoter hypomethylation as a potential epigenetic marker of MDD comorbidity in PD or non-anxious depression, respectively, possibly contributing to a differential pathomechanism of anxiety and mood disorders. In synopsis with previous findings implicating KLF11 in depression-related phenotypes (Harris et al. 2015), this finding is of mechanistic relevance and might foster research efforts into exploring innovative therapeutic approaches targeting this pathway in the treatment of major depression.
